# Facile synthesis of Persian gum–graphene oxide composite as a novel adsorbent for CO_2_ capture: characterization and optimization

**DOI:** 10.1038/s41598-024-56070-w

**Published:** 2024-03-06

**Authors:** Maryam Helmi, Zahra Khoshdouni Farahani, Alireza Hemmati, Ahad Ghaemi

**Affiliations:** 1 School of Chemical, Petroleum and Gas Engineering, Iran University of Science and Technology, Tehran, Iran; 2 Department of Food Science and Technology, Faculty of Agriculture and Food Industry, Science and Research Branch, Islamic Azad University, Tehran, Iran

**Keywords:** CO_2_, Adsorption, Response surface methodology, Persian gum, Thermodynamic, Environmental chemistry, Chemical engineering

## Abstract

Burning fossil fuels releases toxic gases into the environment and has negative effects on it. In this study, Persian gum@Graphene oxide (Pg@GO) was synthesized and used as a novel adsorbent for CO_2_ capture. The characterization of materials was determined through XRD, FTIR, FE-SEM, and TGA analysis. The operating parameters including temperature, Pressure, and adsorbent weight were studied and optimized by response surface methodology via Box–Behnken design (RSM-BBD). The highest amount of CO_2_ adsorption capacity was 4.80 mmol/g, achieved at 300 K and 7.8 bar and 0.4 g of adsorbent weight. To identify the behavior and performance of the Pg@GO, various isotherm and kinetic models were used to fit with the highest correlation coefficient (R^2^) amounts of 0.955 and 0.986, respectively. The results proved that the adsorption of CO_2_ molecules on the adsorbent surface is heterogeneous. Based on thermodynamic results, as the value of ΔG° is − 8.169 at 300 K, the CO_2_ adsorption process is exothermic, and spontaneous.

## Introduction

Today, burning fossil fuels as the main energy source causes increases in toxic gases emotion such as CO_2_, CO, and NO_x_. These toxic gases have negative effects on the environment such as air pollution, photochemical smog, acid rain, etc.^1^. Therefore scientists try to solve these environmental problems using practical and cost-effective technologies to capture materials like toxic gases^2^. Using solid adsorbents such as covalent organic frameworks (COFs), Mg(OH)_2_^3^, DEA solution^4^, graphene oxide (GO), sodium hydroxide (NaOH), and Chitosan is more widely applied because of low equipment corrosion, high-performance good chemical and mechanical properties^5^.

Graphene oxide (GO) is a layer of sp^2^ carbon atoms that is obtained from natural graphite through oxidation^6^. GO contains various oxygenated functional groups^7^ on its basal planes and edges, such as carboxyl, carbonyl, hydroxyl, and epoxy groups^8,9^. These oxygenated functional groups play a significant role in creating either electrostatic interactions or chemical bonding, GO has the potential to serve as a nano-support for biologically active agents, which can be utilized in the creation of innovative catalysts, solid adsorbent, sensors, and drug delivery systems^10^. Furthermore, GO has been explored as a physical adsorbent for harmful greenhouse gases like CO_2_, whose elevated concentrations can pose serious risks to both the environment and human health^11,12^. So, it is need appropriate method for the declining distribution of CO_2_ into the biosphere^13,14^.

The oxygenated functional groups of GO caused alkali in nature. Since the CO_2_ molecules are acidic in nature, the strong interaction between GO and CO_2_ molecules is formed and helps CO_2_ adsorption process^15,16^. Furthermore, due to their hydrophilic nature, GO sheets can be easily integrated into a water-based solution and can be produced as single, double, or multiple layers with exceptional stability. This character makes the both GO surface and its active sites highly effective in improving the capacity of solid adsorption^17^. Although pure GO has limited ability to adsorb CO_2_ and N_2_, its capacity can be enhanced by forming diverse structures.

Bio-polymers like chitin, chitosan, and Persian gum have different advantages including economical, eco-friendly, safe, and suitable approaches. In addition, bio-polymer structures usually have amine groups which enhance the alkali properties of bio-polymer^18^. One of the bio-polymers is Persian gum (Pg). Persian gum is secreted from *Amygdalus scoparia Spach* and is a non-starch hydrocolloid with an acidic polysaccharide nature. The main part of its body consists of the sugar unit of galactose with a smaller amount of arabinose^19^. It is considered in the pharmaceutical, food, textile industries, etc. The resulting solutions cause the medium to become viscous and have emulsifying, texturing, stabilizing, and suspending properties. This gum consists of two insoluble and soluble parts. Iran is known as a good source of Persian gum^20^. The insoluble part of this gum is less used, therefore, in this work, this part is used for synthesis new adsorbent for CO_2_ capture by incorporating with GO.

In this study, Persian gum (Pg) as a biopolymer was used as a part of the solid adsorbent for the CO_2_ adsorption process. By mixing Pg with graphene oxide (GO), Pg@GO was synthesized and used as a novel adsorbent for the CO_2_ adsorption process. Characterization of the adsorbent was studied via X-ray diffraction (XRD), Field Emission Scanning Microscopy (FESEM), Energy Dispersive X-ray (EDX), Fourier transform infrared spectroscopy (FTIR), Thermogravimetric (TGA) analysis. The response surface methodology (RSM) was used to optimize the effect of various independent parameters including temperature, pressure, and adsorbent weight on CO_2_ adsorption capacity. Isotherm, kinetic and thermodynamic models were applied to evaluate the behavior and performance of the adsorption process. Finally, the regeneration of the adsorbent was evaluated at optimum conditions.

## Materials and methods

### Materials

Sulfuric acid (H_2_SO_4_, 98%), nitric acid (HNO_3_, 65%), hydrochloric acid (HCl, 37%), and potassium chloride (KClO_3_, 99%) were collected from Merck company and used without any purification. Carbon dioxide (CO_2_) gas was purchased from Hmtagas company.

### Synthesis GO

The study involved creating GO using a modified version of the Hummers and Offeman method, which overcomes the drawbacks of previous methods and produces high-quality and pure GO^21^. To prepare the GO, two different acids including sulfuric acid and nitric acid were combined in a ratio of 2:1, and graphite powder was added. The mixture was then placed in an ice bath, and potassium chlorate as an oxidation agent was slowly added. After 7 days, the solution turned green. The prepared sample as the oxidized suspension was washed with hydrochloric acid and deionized water to remove impurities and neutralize the sample. Finally, the GO was dried at 60 °C in a vacuum oven.

### Preparation of the insoluble part of Persian gum

For this purpose, a colloidal suspension of Persian gum was prepared. After an overnight, the insoluble part is separated and another solution is prepared, and this process is repeated three times. Finally, the separated insoluble part was dried in an oven and its particle size was reduced by grinding and meshing^20^.

### Synthesis of Pg@GO adsorbent

**Figure 1:**
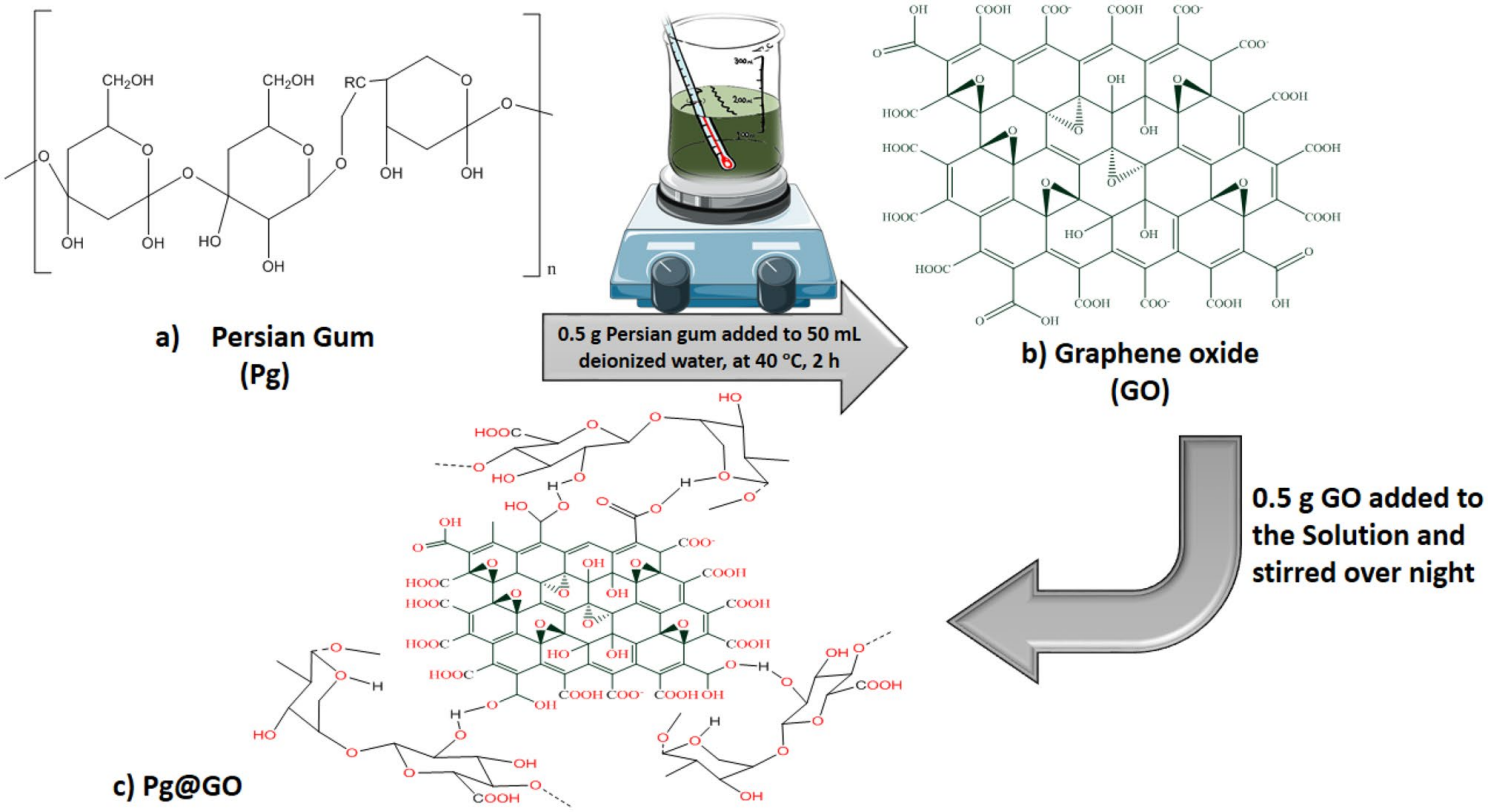
The steps of the synthesis of Pg@GO as a solid adsorbent.

To synthesis Pg@GO as a solid adsorbent, first, 0.5 g of Persian gum was dissolved in 50 mL deionized water for 2 h at 40 °C until a homogenous solution was achieved. Then, 0.5 g GO was added to the solution and stirred over the night. Next, the solution was dried at room temperature for two weeks. Finally, the prepared sample was used as a solid adsorbent for CO_2_ adsorption. Figure 1 shows each step of synthesis of Pg@GO as a solid adsorbent.

### CO_2_ adsorption setup

**Figure 2:**
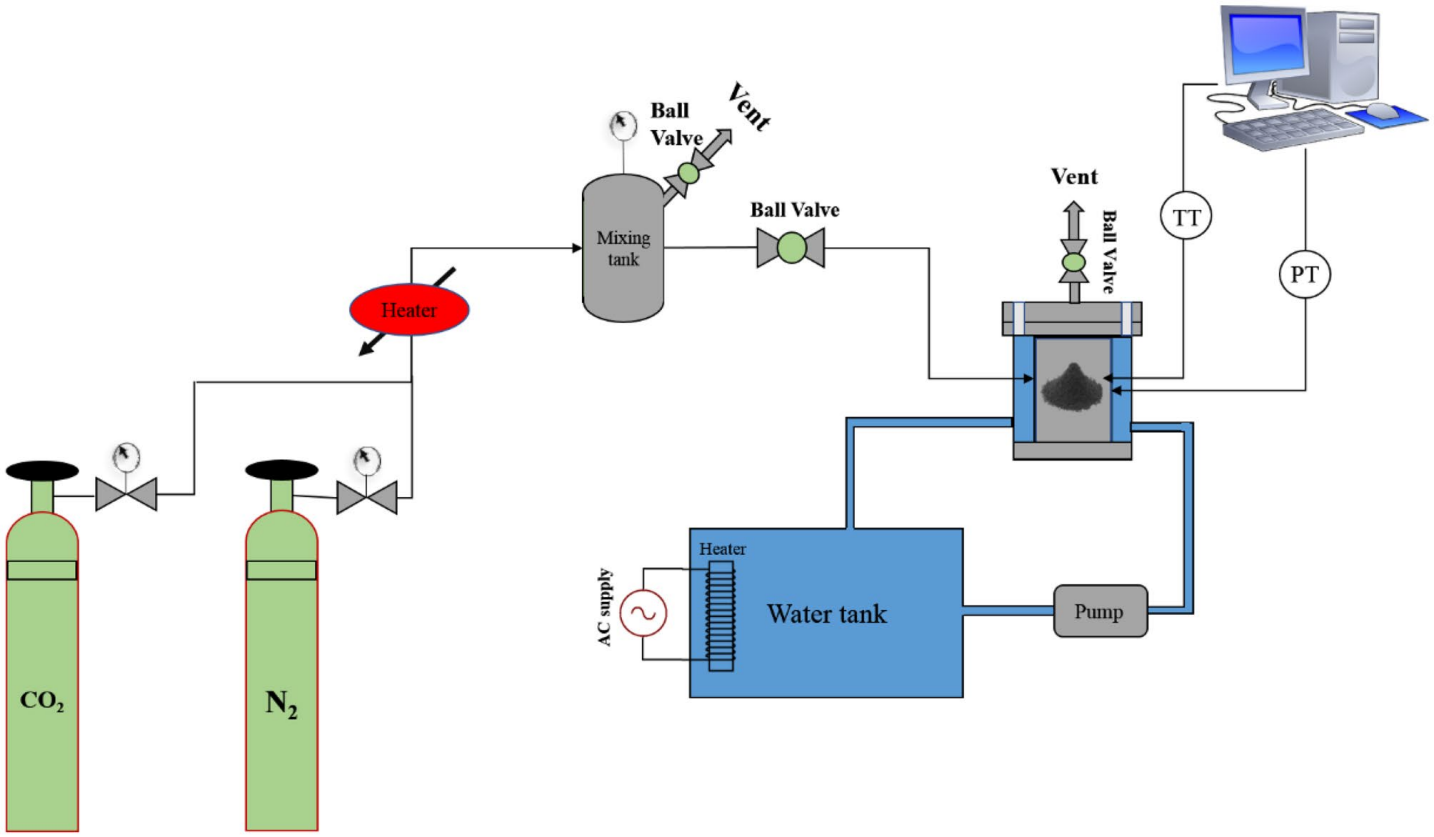
CO_2_ adsorption setup.

The laboratory-scale reactor depicted in Fig. 2 was utilized for the CO_2_ adsorption process and consisted of four main parts: gas injection, a reactor system, an instrument for controlling CO_2_ pressure variations during the adsorption process, and a thermocouple for monitoring test heat. During the adsorption process, CO_2_ gas flowed from a high-purity capsule into the chamber, with different weights of solid adsorbent used in each run. As the adsorption process commenced, the chamber's pressure decreased due to CO_2_ adsorption, as the volume of both the chamber and reactor remained constant. The amount of CO_2_ adsorbed was calculated based on the pressure reduction. In another word, when unadulterated CO_2_ was implanted into the system, the adsorption operation was as takes after. The CO_2_ gas stream was traded from the capsule to the chamber containing the specified adsorbent inside the reactor. At that point, the adsorption started, and CO_2_ was ingested by the adsorbent. Due to the steady volume of the reactor and the chamber, the CO_2_ adsorption decreased weight inside the chamber, and concurring to the entirety of weight diminish utilizing conditions related to adsorption, the amount of CO_2_ adsorption (%) and the value of CO_2_ adsorbed were measured by Eqs. (1) and (2):

1 $${\text{Adsorption}}(\% ) = \left( {{\raise0.7ex\hbox{${p_{i} - p_{f} }$} \!\mathord{\left/ {\vphantom {{p_{i} - p_{f} } {P_{i} }}}\right.\kern-0pt} \!\lower0.7ex\hbox{${P_{i} }$}}} \right) \times 100$$

2 $$n_{{{\text{CO}}_{2} }} = {\raise0.7ex\hbox{${\left( {P_{i} - P_{f} } \right)V}$} \!\mathord{\left/ {\vphantom {{\left( {P_{i} - P_{f} } \right)V} {RTZ}}}\right.\kern-0pt} \!\lower0.7ex\hbox{${RTZ}$}}$$

where *p*_*i*_ is initial pressure, *p*_*f*_ is final pressure. *w* is the weight of the adsorbent. In addition, the adsorption capacity of adsorbent was measured by Eq. (3)^22^:

3 $$q_{e} = \left( {{\raise0.7ex\hbox{${(p_{i} - p_{{_{f} }} )VM_{{{\text{CO}}_{2} }} }$} \!\mathord{\left/ {\vphantom {{(p_{i} - p_{{_{f} }} )VM_{{{\text{CO}}_{2} }} } {RTZ}}}\right.\kern-0pt} \!\lower0.7ex\hbox{${RTZ}$}}} \right) \times 10^{3}$$

where *V* is the reactor volume, *M*_CO2_ is 44 g/mol, and *R* is the gas constant.

### Characterization of adsorbent

The synthesis of Pg@GO was studied by various analysis methods to check structure, morphology, and composition. The structures and surface features of solid the adsorbent was examined using Scanning Electron Microscopy (SEM, Philips XL30 ESEM). X-ray power diffraction (XRD, Philips PW1730) was used to analyze the characteristics and recognition of a crystalline catalyst. The XRD result was recorded for a period of 2 h, covering a range of 5–70° with an increment of 0.05° and a duration of 1 s per step. The Brunauer–Emmett–Teller (BJH, BELSORP MINI II, and BEL) was utilized to identify the shapes and surface areas of non-porous adsorbents and a mesoporous solid with varying pore sizes. It means that the results of BET analysis assist in understanding adsorbent is macroporous (> 50 nm) or mesoporous (2–50 nm) or microporous (< 2 nm)^23^. The Fourier Transform Infrared spectroscopy (FTIR, Thermo, Avatar) provided information on the molecular vibrations of the catalysts within the 400–4000 cm-1 range. Data on the thermal stability of Pg@GO was obtained using Thermo gravimetric analysis (TGA, TA, Q600, USA) by measuring the change in weight of the adsorbent as it is heated at a constant rate.

### Experimental design

To improve the process of CO_2_ adsorption by Pg@GO, the response surface methodology (RSM) based on the Box–Behnken design (BBD) was utilized. RSM-BBD as an experimental design statistical tool was applied to specific interactions between two independent parameters and optimized response of multiple parameter processes. Alhajabdalla et al.^24^ reported the main benefit of RSM-BBD. RSM-BBD ability is to analyze independent variables by performing few experimental runs than other RSM methods^25^. The study focused on three independent factors: pressure, time, and adsorbent weight. The dependent factor is CO_2_ adsorption capacity. Table 1 provides the design and range levels for each parameter. To determine the absolute errors, seventeen experiments with six duplicate runs were conducted at central points, including 16 factorial points, six central, and eight axial^26^. The results of these experiments are presented in Table 2.

4 $$q_{{{\text{CO}}2}} = \alpha_{0} + \times \sum\limits_{i = 1}^{k} {\alpha_{ii} X_{i} + \sum\limits_{i = 1}^{k} {\alpha_{ii} X_{i}^{2} + } \sum\limits_{i - 1}^{2} {\sum\limits_{j = 1 + 1}^{3} {\alpha_{ij} X_{i} X_{j} + \varepsilon } } }$$

**Table 1:** Experimental design levels of factors according to BBD.

Factors	Units	symbol	Levels		
			− 1	0	+ 1
Adsorbent weight	g	A	0.1	0.3	0.5
Pressure	bar	B	1	4.5	8
Temperature	°C	C	25	40	55

**Table 2:** Experimental results for CO_2_ adsorption capacity obtained from BBD.

Run	A: adsorbent weight	B: pressure	C: temperature	Adsorption capacity
	g	P	°C	mmol/g
1	0.5	4.5	55	3.49
2	0.1	1.0	40	1.95
3	0.5	4.5	25	4.30
4	0.3	1.0	25	2.81
5	0.3	4.5	40	3.07
6	0.5	8.0	40	4.74
7	0.3	1.0	55	1.76
8	0.3	4.5	40	3.00
9	0.1	4.5	25	3.61
10	0.3	8.0	55	3.04
11	0.1	8.0	40	3.43
12	0.3	4.5	40	3.02
13	0.3	8.0	25	4.68
14	0.3	4.5	40	3.06
15	0.1	4.5	55	1.60
16	0.5	1.0	40	3.12
17	0.3	4.5	40	3.04

The value of q_CO2_, which represents the CO_2_ adsorbent capacity, is determined by the coefficients α_0_, αi, α_ii_, and α_ij_ calculated through regression programming. These coefficients represent the constant, linear, quadratic, and interaction factors. *X*_*i*_ and *X*_*j*_ are independent variables, while ε represents error^27,28^.

## Result and discussion

### Structure properties of Pg@GO

**Figure 3:**
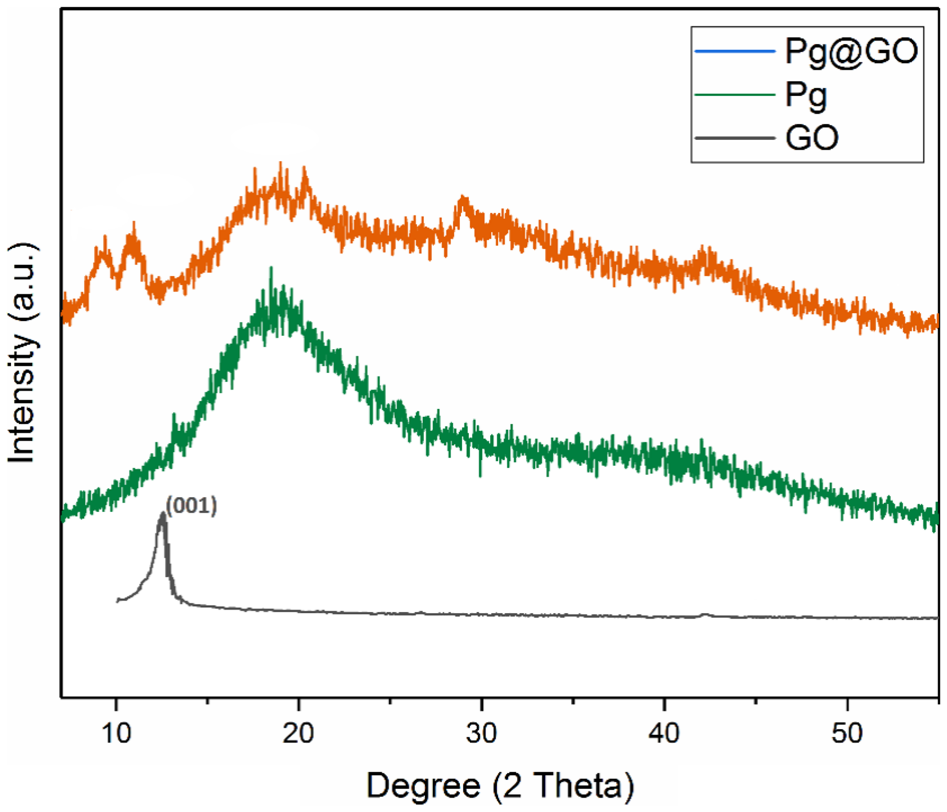
XRD patterns for raw GO, raw Persian gum, Pg@GO.

**Figure 4:**
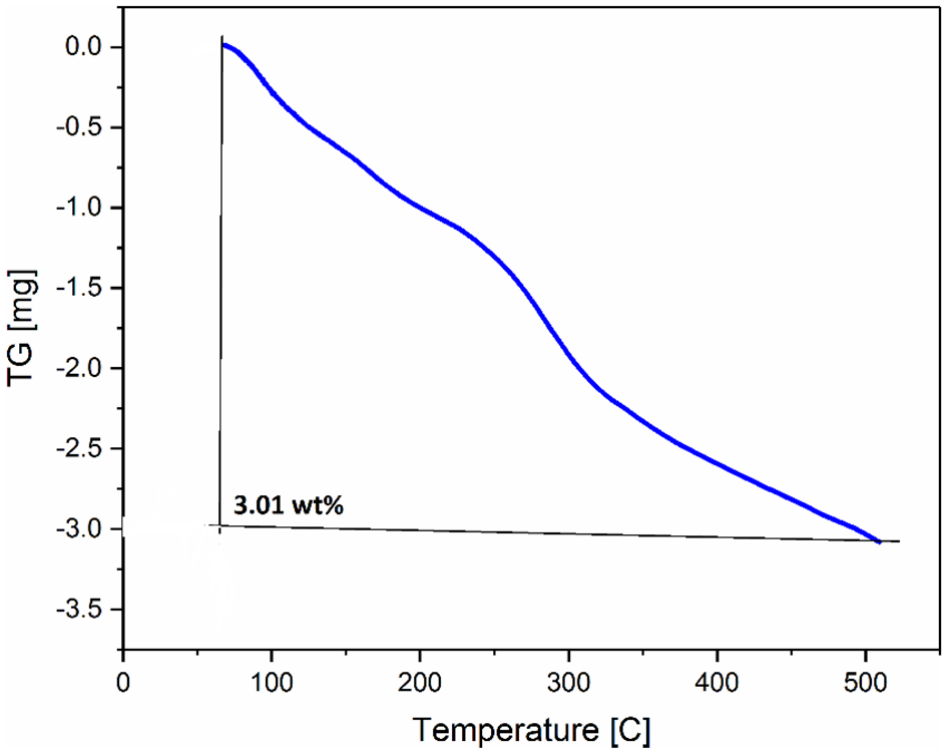
TGA analysis for Pg@GO as a solid adsorbent.

The crystal structure of raw Persian gum (Pg), GO and Pg@GO was studied by XRD analysis and the results are shown in Fig. 3. Persian gum has board diffraction peaks at 20–42° which shows semi-crystal micro-structure of Pg@GO^29^. Bashir and Haripriya reported that lacking of sharp peaks in the XRD pattern of Pg showed its amorphous character^29^. The diffraction peak at GO appeared 2θ of 10.01°, a crystal plane with a d-value of 8.75 A° correspond to the typical diffraction peaks of GO sheets^30^. The XRD pattern of Pg@GO has main peaks at 9.22°, 10.20°, and 20° related to the crystalline structure of GO and Pg. The accessibility of these peaks shows that the synthesis of Pg@GO was successful, however, the intensity of GO peak at 9.22° decreases. The thermal stability of Pg@GO was determined by TGA analysis and showed three stages of the weight loss (Fig. 4). The first step at < 100 °C related to the loss of adsorbent water and moisture. It was increasing temperature from 100 to 360 °C related to the thermal decomposition of oxygen-containing groups in GO and Persian gum to CO_2_, CO, and H_2_O^31,32^. The third stage occurs at 360 to 1000 °C because of oxidative pyrolysis of the carbon frame work of GO after the elimination of the oxygen-containing groups^33^.

**Figure 5:**
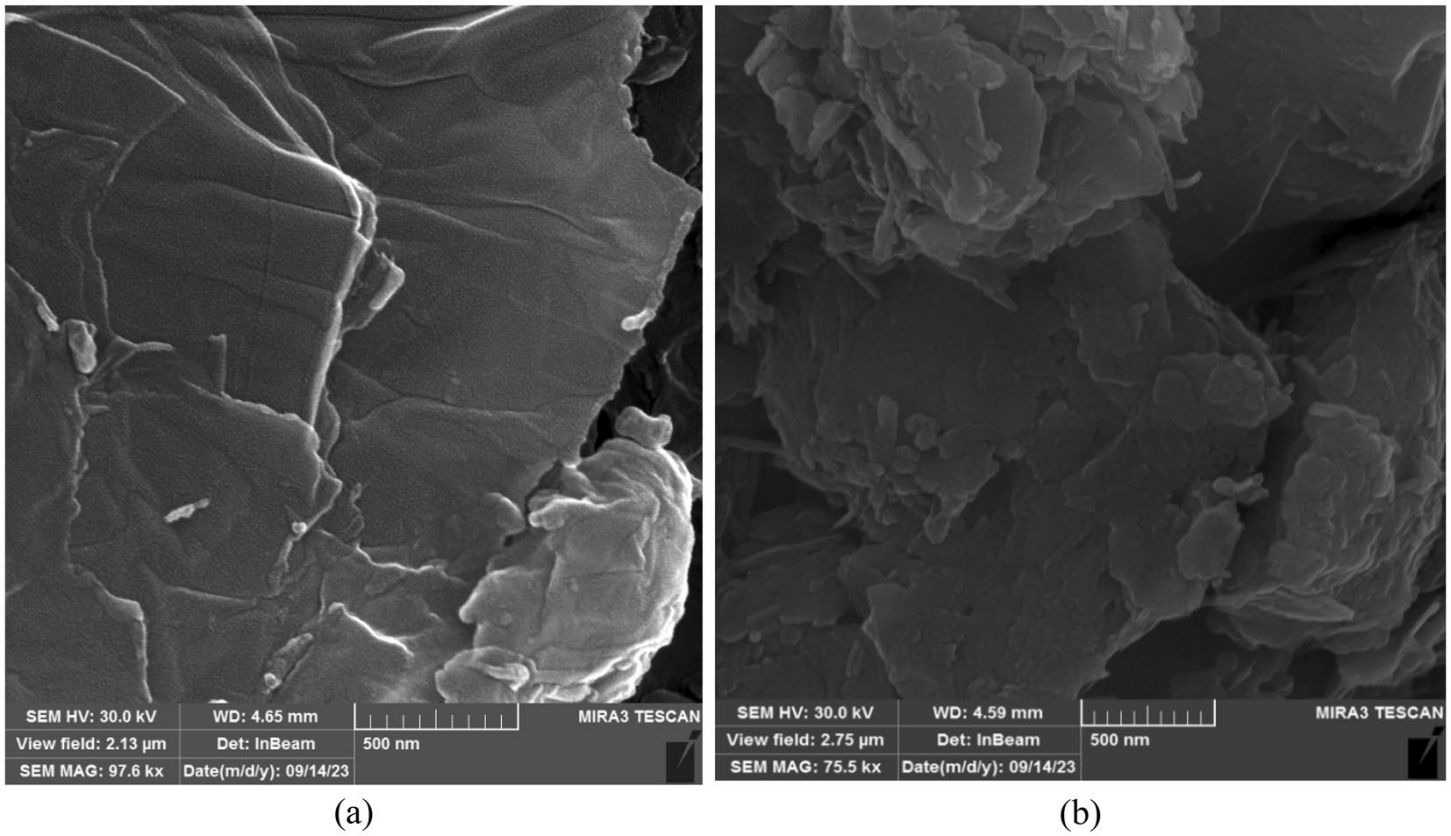
FESEM images for (**a**) raw GO, (**b**) Pg@GO.

The morphology structure of raw GO and Pg@GO was studied by FESEM analysis, and the result is shown in Fig. 5. The flat morphology of GO is shown in Fig. 5a, and it has oxygen functional groups that cause the surface of GO seem smooth and wrinkled^34^. GO’s thin layers structure displays that GO has a folded and rippled wavy shape. The oxidation process causes the edges of the exfoliated GO to crumple^35^. The unique structure of GO induced its porous location on the sheet or between sheets^36^. Based on Fig. 5b, after the immobilization of Persian gum on GO, the surface of GO creates a surface remarkably rougher^37^. Also, as Pg has a soft, and free pores structure^38^, Pg@GO has low amount of pores in its structure.

**Figure 6:**
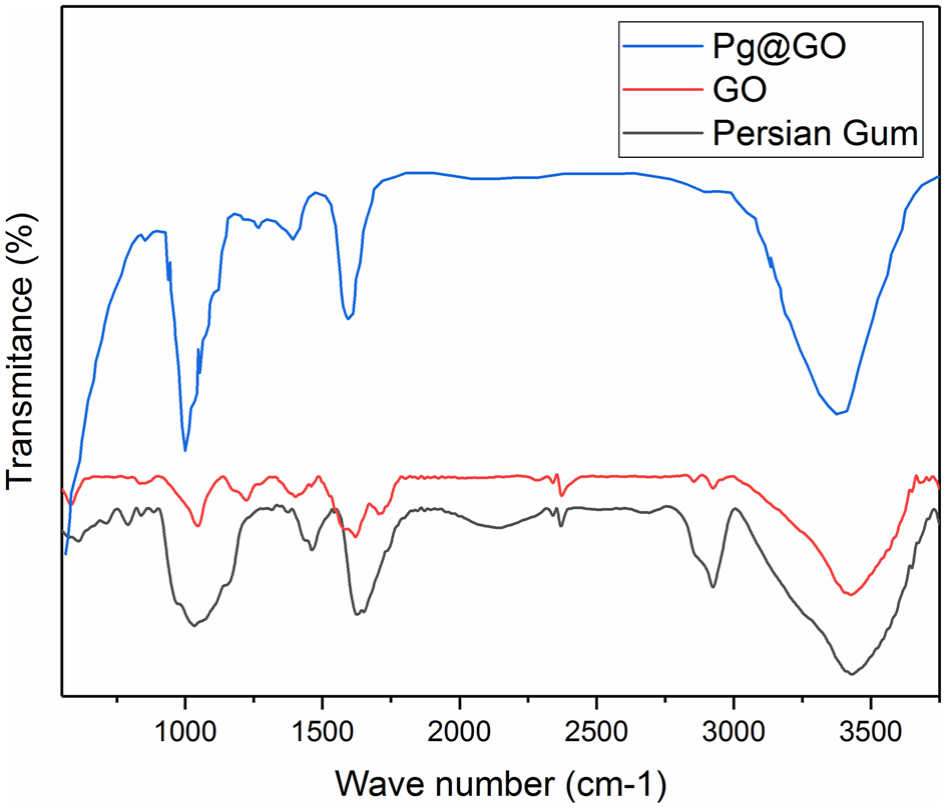
FTIR spectrum for raw GO, pure Persian gum, and GO@Pg.

The functional groups of raw GO, Persian gum, and Pg@GO as adsorbent were determined by FTIR analysis, and its results are shown in Fig. 6. The board peaks at 3200–3400 cm^−1^ which can be related to –OH stretching vibration. The characteristic peaks at 1035 cm^−1^ and 1167 cm^−1^ are related to either epoxy or alkoxy (C–O), and stretching vibration of C–O, respectively. The peak at 1415 cm^−1^ relates to the carboxy (C–O). The peak at 1720 is related to the carboxylic acid (C=O)^39^. The FTIR spectra of raw Persian gum have a board peaks at 3300–3400 cm^−1^ showed –OH stretching vibration. The main peaks at 2927, 2926, and 2924 cm^−1^ are related to the asymmetric –CH_2_– functional group. The peak at 2855 cm^−1^ corresponds to the symmetric stretching vibrations of the –CH_2_– functional group. The peaks at 1601 and 1602 cm^−1^ are assigned to the asymmetrical stretching of carboxylate groups or the intramolecularly bound water. The amid I in protein has peaks at 1600 and 1700 cm^−1^. The stretching vibration of alcoholic groups creates peaks at 1023 and 1024 cm^−1^. The carbohydrate fingerprint has peaks at 1500 and 500 cm^−1^^40,41^. The FTIR spectra of Pg@GO has characteristic bands of Persian gum on the solid adsorbent which show the presence of Persian gum species without any ruin after loading on GO. However, the characteristic bands of Pg@GO are displacement and become visible very weaker comparing the FTIR spectra of the raw Persian gum.

### Adsorbent mechanism

**Figure 7:**
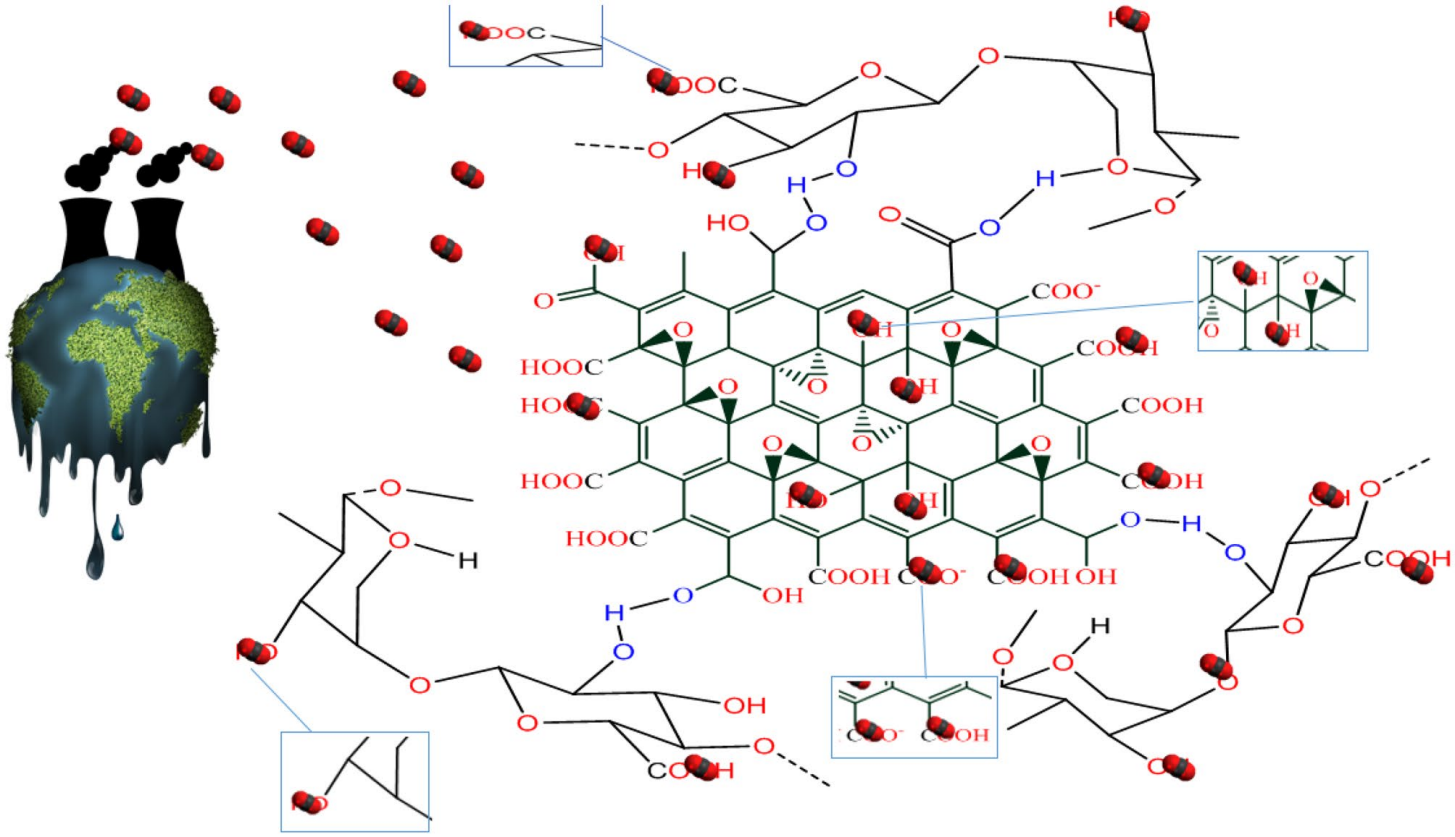
Proposed mechanism for adsorption of CO_2_ molecules using Pg@GO.

The results showed that in low temperatures, the adsorption of CO_2_ on Pg@GO was physisorption. Both GO and Persian gum have hexagonal structures with hydroxyl, epoxyl, and carbonyl groups^42,43^. The interaction between CO_2_ molecules and the delocalized π-aromatic system of GO are seen because of the presence of oxygen groups in the interlayers. Based on the different reports, the high amount of oxygen groups on Pg@GO has a positive effect on CO_2_ adsorption. As both Persian gum and GO have oxygen functional groups on their chemical structures, CO_2_ adsorption capacity was performed with increasing pressures^9,11,44^. Figure 7 shows the suggested mechanism for CO_2_ adsorption via Pg@GO.

### Adsorption modeling

#### Isotherm modeling

Adsorption isotherm is used for understating how CO_2_ molecules as adsorbed interact with the active sites of adsorbent in a variation of gas pressures at constant temperature^45,46^. This interaction usually is determined by adsorption isotherm models including Langmuir, Freundlich, Dubinin–Radushkevich (D–R), Sips, and Temkin. Equation (5) shows Langmuir isotherm model. Based on Langmuir model as a simple and empirical model, the rate of adsorption and desorption of CO_2_ molecules on solid surface is equal. The adsorption process happens in limited specific sites and adsorb only one molecule^47^.

5 $$q_{e} = \frac{{q_{m} K_{L} P_{{CO_{2} }} }}{{1 + K_{L} P_{{CO_{2} }} }}$$

where *q*_*e*_ is the maximum adsorption capacity (mmol g^−1^), *q*_*m*_ is the amount of CO_2_ absorbed at equilibrium (mg g^−1^), and *K*_*L*_ is constant Langmuir (bar^−1^). *P*_*CO2*_ is the equilibrium pressure of the gas. The Freundlich model is calculated based on Eq. (6). This model is used for inhomogeneous and homogenous surfaces. It can use for chemical and physical adsorption^48^.

6 $$q_{e} = K_{F} P^{{{\raise0.7ex\hbox{$1$} \!\mathord{\left/ {\vphantom {1 n}}\right.\kern-0pt} \!\lower0.7ex\hbox{$n$}}}}$$

*K*_*f*_ and *is* the Freundlich constant (g bar mmol^−1^).

Dubinin–Radushkevich model (D–R) is applied for the recognition of either chemical or physical sorption^48^. D–R model is calculated by Eq. (7).

7 $$\ln q_{e} = \ln q_{m} - \beta .\varepsilon^{2}$$

where *q*_*m*_ shows a single-layer adsorption capacity, *β* and *ε* are the constant related to the adsorption energy (mol^2^ KJ^−2^). The sign of *ε* or the Polanyi potential (KJ^2^ mol^−2^) is calculated by Eq. (8):

8 $$\varepsilon = RT\ln \left[ {1 + \frac{1}{{P_{{CO_{2} }} }}} \right]$$

where *R* is gas constant (J mol^−1^ K^−1^), and *T* is the absolute temperature (k). Temkin isotherm model is determined by Eq. (9). According to the Temkin isotherm assumption, the adsorption heat related to the adsorption process correlated to all adsorbent molecules and reduced linearly rather than logarithmic with coverage^49^.

9 $$q_{e} = B\ln (K_{T} p)$$

In this equation, *B*, and* K*_*T*_ (atm^−1^) are Temkin constant. By mixing Langmuir and Freundlich isotherm models, the Sips model is obtained, while the main difference between Sips and Langmuir models is *n*_*s*_. The *n*_*s*_ is the heterogeneity parameter. Usually, it is lower than 1 which shows more heterogeneity of the surface of adsorbents. If *n*_*s*_ is equal to 1, the adsorbent surface is homogenous, and the Sips model reduces to Langmuir model^50^. Sip isotherm model is determined by Eq. (10).

10 $$\begin{gathered} b_{s} = b_{0} .\exp \left( {\frac{Q}{{R.T_{0} }}} \right).\left( {\frac{{T_{0} }}{T} - 1} \right);n_{s} = n_{0} + \alpha \left( {1 - \frac{{T_{0} }}{T}} \right); \hfill \\ q_{ms} = q_{m0} .\exp (\chi (1 - \frac{T}{{T_{0} }})) \hfill \\ \end{gathered}$$

where *b*_0_* is* adsorption affinity, *n*_0_ is heterogeneity coefficient*, q*_*m*0_* describes* maximum adsorption capacity, *R* and *T*_0_ are gas constant and reference temperature which assumed 298 K, respectively. Two signs including *χ* and α are Sips constants.

**Figure 8:**
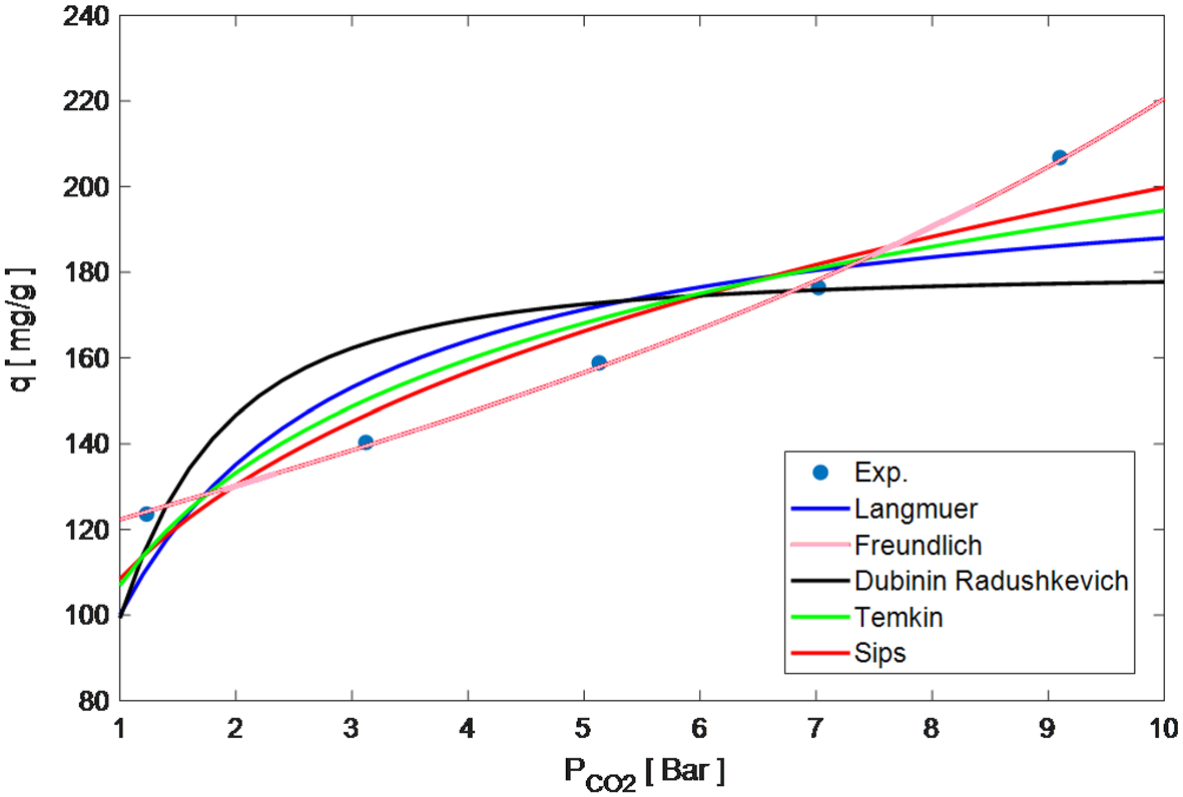
Isotherm modelling of CO_2_ adsorption on Pg@GO at 298 K.

Figure 8, and Table 3 display the CO_2_ adsorption isotherm models. The isotherm experiments were performed at constant temperature of 298 K and at pressure in range of 1–9 bar. For each isotherm model, the high value of R^2^ proves which isotherm model is well fitted to experimental data. According to Table 3, the Sips model has a high value of R^2^ and has the highest accuracy. This means that CO_2_ adsorption process was carried out heterogeneous and multi-layered on Pg@GO.

**Table 3:** Parameters of isotherm models for CO_2_ adsorption process at 25 °C.

Isotherm models	Parameters		Value
Langmuir	q_m_ (mg/g)		208.346
	k_l_		0.925
	R^2^		0.876
Freundlich	K		108.469
	n		3.769
	R^2^		0.955
Dubinin Radushkevich	q_s_		179.775
	β		0.208
	E_a_		1.550
	R^2^		0.777
Temkin	A		16.684
	B		38.006
	R^2^		0.931
Sips	K_s_		− 1513.319
	β		− 0.813
	a_s_		− 13.36731
	R^2^		0.999

#### Kinetic modeling

Both the adsorption rate and mechanism are calculated by adsorption kinetic^51,52^. In this study, four nonlinear kinetic models are used and they are listed in Table 4. The adsorption rate based on adsorption capacity is known as a first-order equation. This phenomenon is seen when the adsorption process happens in different layer by diffusion^53^.

11 $$q_{t} = q_{e} (1 - \exp ( - k_{1} .t))$$

where *q*_*t*_, *q*_*e,*_ and *K*_1_ are adsorption capacity at time (mg g^−1^), equilibrium condition (mg g^−1^), and first order rate constant (min^−1^), respectively. Another kinetic model is a second-order kinetic model that is based on solid-phase adsorption. It shows that the rate of chemical adsorption is slow and it can control adsorption process^51,54^. Equation (12) is used for determining second-order kinetic model.

12 $$q_{t} = K_{2} \cdot q_{e}^{2} \frac{t}{{(1 + K_{2} \cdot q_{e} \cdot t)}}$$

where *K*_2_ is second order rate constant (min^−1^), and* t* is adsorption time (min). The theory of Ritchie’s second-order equation (Eq. 13) is that each adsorption process is performed on two surface sites^47^.

13 $$q_{t} = q_{e} - \frac{{q_{e} }}{{(1 + K_{2} t)}}$$

**Table 4:** Kinetic parameters of CO_2_ adsorption on Pg@GO at different temperature.

Kinetic models	Parameters	25 °C	35 °C	45 °C	55 °C	65 °C
First order	q_e_ (mg/g)	173.631	173.809	154.185	113.893	116.918
	k_l_	0.01320	0.01073	0.02253	0.00433	0.00237
	R^2^	0.859	0.839	0.941	0.838	0.927
Second order	q_e_ (mg/g)	181.761	183.098	158.318	121.407	127.652
	k_l_	0.00010	0.00008	0.00025	0.00006	0.00003
	R^2^	0.931	0.921	0.972	0.909	0.963
Ritchie second order	q_e_ (mg/g)	181.761	183.098	158.418	121.407	127.652
	k_l_	0.01870	0.01490	0.03944	0.0067	0.0034
	R^2^	0.931	0.921	0.972	0.909	0.963
Elovich	α	1.991	0.490	5210.568	0.034	0.006
	β	15.753	17.773	8.467	15.670	19.628
	R^2^	0.961	0.967	0.959	0.959	0.986

The Elovich model is suggested for chemical adsorption process^55^.

14 $$q_{t} = \beta \cdot \log (\alpha \cdot \beta ) + \beta \cdot \log (t)$$

**Figure 9:**
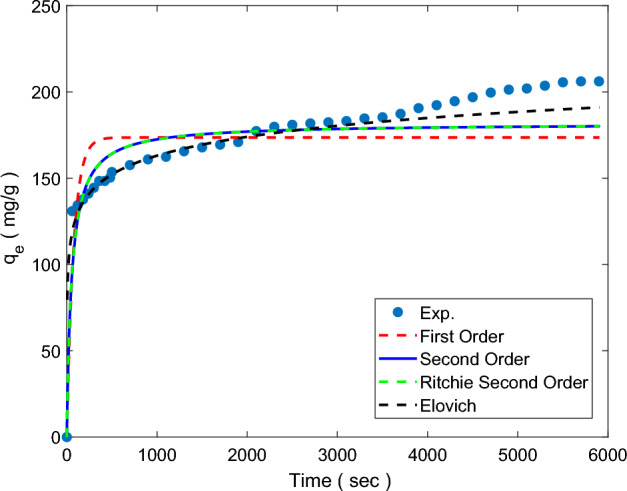
Kinetic modelling of CO_2_ adsorption on Pg@GO at different pressures.

α and β are the initial adsorption rate (mg g^−1^ min^−1^), and the value dependent on the activation energy and amount of surface coverage (g mg^−1^), respectively. Parameters related to the different kinetic models that are used in this study for Pg@GO are listed in Table 4. Figure 9 shows CO_2_ adsorption capacity (mg/g) against time (sec). Based on Fig. 9, the highest value of R^2^ is related to the Elovich model which shows this model is best fitted to the empirical data, and then the second-order model has a high value of R^2^. Elovich's model proves that the CO_2_ capture process is not constant during the adsorption process and Pg@GO has a uniform surface because surface coverage increased hence adsorption rate decreased, and the uniform surface area of the adsorbent causes the active sites for CO_2_ adsorption are not constant.

#### Thermodynamic modeling

**Figure 10:**
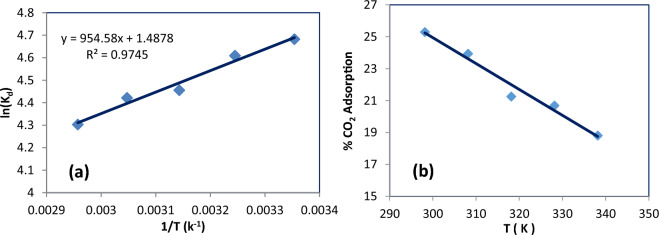
CO_2_ captures (**a**) Van’t Hoff plot. (**b**) In different temperatures.

The properties of the CO_2_ adsorption mechanism were investigated by thermodynamic factors of enthalpy change (ΔH°), change in entropy (ΔS°), and Gibbs free energy in change (ΔG°) in physical and chemical adsorption. The parameters and magnitude are very important. In other words, the negative value of ΔH° shows an exothermic reaction, in contrast, the positive value ΔH° illustrates an endothermic reaction. In addition, if ΔH° is lower than 20 kJ/mol, absolute physisorption will happen in the process, while if ΔH° is more than 40 kJ/mol chemical adsorption will accrue^4,56^. The value of randomness of adsorption capture in the organization at the interface of gas/solid is determined by the positive and the negative signs of ΔS°. On one hand, when ΔS° is more than zero (ΔS° > 0), the process is more random. On the other hand, if ΔS° is lower than zero (ΔS° < 0), the process is less random. Moreover, the spontaneity of the process determines with signs of the Gibbs free energy change^57^. If the ΔG° is more than zero (ΔG° > 0), the process is not happen and is nonspontaneous, conversely, the negative value of ΔG° (ΔG° < 0), the process is possible and spontaneous, according to Fig. 10a, CO_2_ adsorption process via Pg@GO was possible and spontaneity^58,59^. The following equations assist thermodynamic factors to be calculated. The thermal adsorption enthalpy is calculated by Van’t Hoff equation (Eq. 17), which was obtained by Eqs. (15) and (16). K_d_ as the distribution coefficient calculated by Eq. (18).

15 $$\Delta G^{0} = \Delta H^{0} - T\Delta S$$

16 $$\Delta G^{0} = - RT\ln K_{d}$$

17 $$\ln K_{d} = \frac{{\Delta S^{0} }}{R} - \frac{{\Delta H^{0} }}{RT}$$

18 $$K_{d} = (P_{i} - P_{e} ) \times (V/W)$$

where T and R are absolute temperature and the value of gas constant (8.314 J mol^−1^ K^−1^), respectively. When ln K_d_ against 1/T was plotted the values of ΔH° (slop) and ΔS° (intercept) were calculated. However, the value of ΔG° is measured by Eq. (12). K_d_ is the distribution coefficient value calculated by Eq. (15). P_i_ (bar) is the initial pressure, *P*_*e*_ (bar) is the reduced pressure, V (cm^3^), and W (g) are, the volume, and weight of the adsorbent, respectively.

Based on Fig. 10b, and Table 5, temperature has a significant effect on CO_2_ adsorption. When temperature increases, CO_2_ adsorption decreases. Rashidi et al.^60^ reported that since the type of bonds between CO_2_ molecules and solid adsorbents were van der Waals as weak bonds, increasing temperature cause these bonds destroyed. As solid adsorbent faced High CO_2_ molecules penetration, large surface adsorption energy, and instability of adsorption CO_2_ molecules to the solid adsorbent surface, the desorption process by Le Chatelier’s principle is an endothermic type and suitable.

**Table 5:** Thermodynamic variables for CO_2_ adsorption on Pg@GO adsorbent.

P(CO_2_) Bar	ΔH (kJ/mol)	ΔS (kJ/mole K)	ΔG (kJ/mol)				
			25	35	45	55	65
6.000	− 11.441	− 0.011	− 8.169	− 8.059	− 7.949	− 7.83949	− 7.730

### RSM statistical analysis

RSM-BBD was used to optimize the CO_2_ adsorption process on Pg@GO. Based on the BBD, the experimental design was used to extend an RSM by the Quadratic model. Three independent factors such as Adsorbent weight (A), Pressure (B), and Temperature (C), was used in the RSM model while the adsorbent capacity (Y) was used as response. The quadratic equation on the design model was shown in Eq. (19). On the below equation negative coefficient will decline the CO_2_ adsorption capacity, while the positive equation will increase the CO_2_ adsorption capacity.

19 $$q_{{{\text{CO}}_{2} }} = 3.04 + 0.63A + 0.78B - 0.69C + 0.30AC - 0.15BC + 0.22A^{2} + 0.047B^{2}$$

To specify the coefficients of the quadratic of the empirical results, Analysis Of Variance (ANOVA) was applied. The coefficient of determination (R^2^) was used to calculate the accuracy of the suggested model, and then F-test was applied to check statistical significance^61,62^. According to Table 6, the quadratic model has an F-value equal to 1404.32 low error probability value ((Prob > F) < 0.0001) displayed that the mathematical models can be statistically illustrating the achieved experimental results. The F-value is obtained by dividing two mean squares, which allows for the assessment of the ratio between explained variance and unexplained variance. The calculation of the *p*-value is dependent on the sampling distribution of the test statistic assuming the null hypothesis, the data collected from the sample, and the specific type of test conducted (lower-tailed, upper-tailed, or two-sided)^63^.

**Table 6:** ANOVA for Response Surface Quadratic model.

Source	Some of Squares	Df	Mean square	F value	*p*-Value Prob > F	
Model	12.56	9	1.40	1404.32	< 0.0001	Significant
A-Adsorbent weight	3.20	1	3.20	3221.16	< 0.0001	Significant
B-Pressure	4.88	1	4.88	4914.41	< 0.0001	Significant
C-temperature	3.80	1	3.80	3819.57	< 0.0001	Significant
AB	4.900E−003	1	4.900E−003	4.93	0.0618	Not-significant
AC	0.36	1	0.36	362.33	< 0.0001	Significant
BC	0.087	1	0.087	87.59	< 0.0001	Significant
A^2^	0.21	1	0.21	214.06	< 0.0001	Significant
B^2^	9.400E−003	1	9.400E−003	9.46	0.0179	
C^2^	6.845E−004	1	6.845E−004	0.69	0.4339	
Residual	6.955E−003	7	9.936E−004			
Lack of Fit	3.675E−003	3	1.225E−003	1.49	0.3442	Not significant
Pure error	3.280E−003	4	8.200E−004			
Cor total	12.56	16				
R^2^	0.9994		Std. dev	0.032		
Pre-R^2^	0.9949		Mean	3.16		
Adj-R^2^	0.9987		C.V%	1.00		

Where A, B and C are codded form of independent factors. Interaction terms are AC, BC, and AB, and squared terms of factors are described by A^2^, B^2^ and C^2^.

Since the R^2^ value was 0.9994, therefore, experimental results have good agreement with the model. The significance of each factor was introduced by *p*-value and F-test. The smaller *p*-value and larger F-test show the significant effects of independent factors^64^. It was clear that the order of priority among those factors on the CO_2_ adsorption capacity is the quadratic terms of adsorbent weight (F = 3221.16), pressure (F = 4914.41), and temperature (3819.57). The interaction effect between factors, however, has less impact on the adsorption process.

#### The effect of independent factors

**Figure 11:**
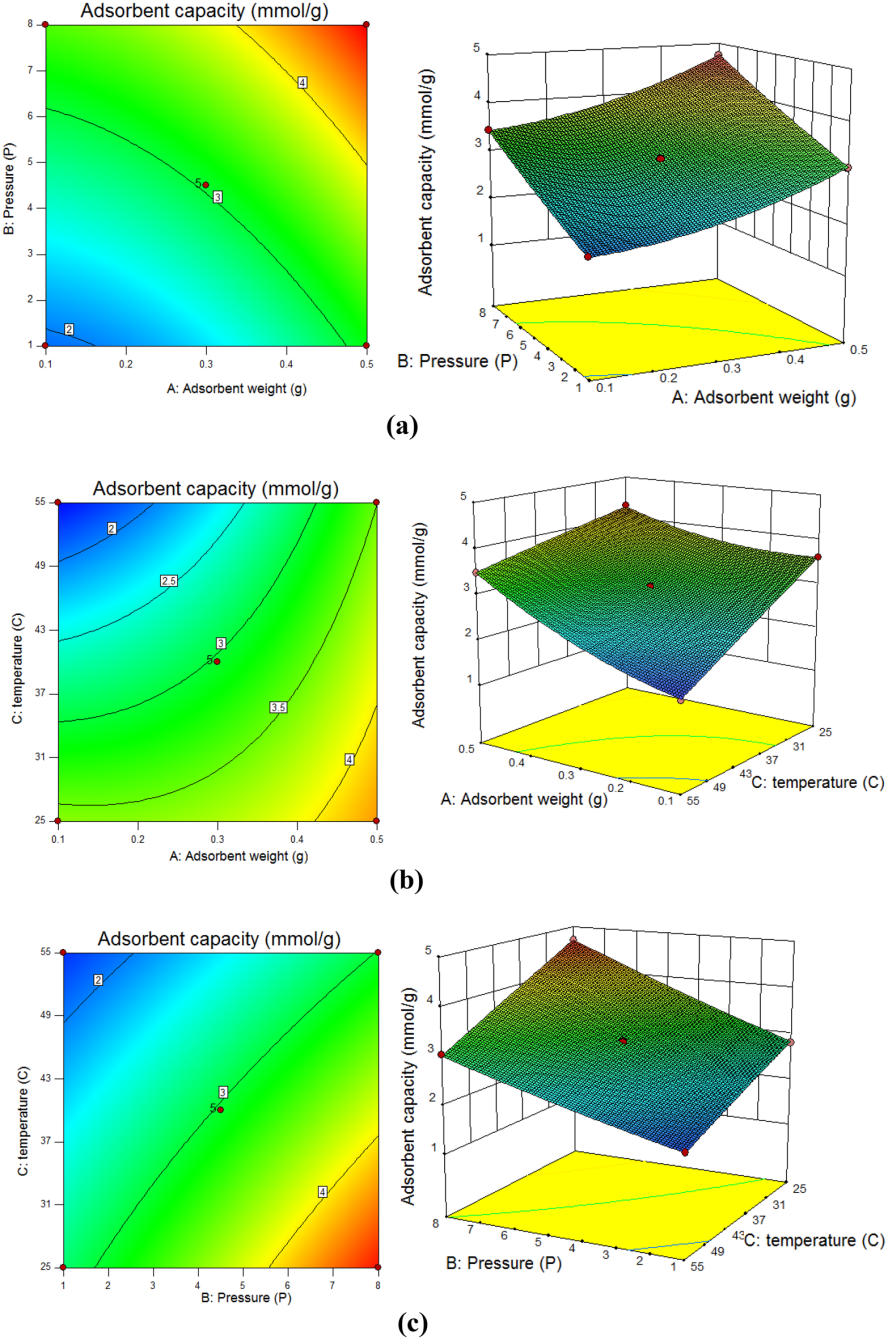
The interaction between (**a**) adsorbent weight and pressure factors, (**b**) adsorbent weight and temperature factors, and (**c**) pressure and temperature factors.

Figure 11 shows theinteraction between independent factors. Figure 11a shows the interaction between adsorbent weights (A) and pressure (b), while temperature (C) is considered constant. According to the results, by increasing the adsorbent weight to 0.4 g and pressure to 7.88 bar, the CO_2_ adsorption capacity was raised to 4.80 mmol/g. That is because the interaction between active sites of solid adsorbent and CO_2_ molecules increased significantly. Therefore, the yield of CO_2_ adsorption capacity improved^65^. The growing active sites decrease adequate space for CO_2_ capture, therefore, both agglomeration and closure of pores are observed^66^. According to the mentioned reasons 0.4 g adsorbent weight was chosen.

Figure 11b illustrates the interaction between adsorbent weight (A) and temperature (C). It shows that, by increasing temperature and adsorbent weight to 27 C and 0.4 g, CO_2_ adsorption capacity improved to 4.80 mmol/g. This phenomenon happened since CO_2_ adsorption is exothermic in nature, by increasing temperature the interaction between active sites and gas molecules augments, because of this molecular interaction grows and accessible and effective active sites on the surface of adsorption decline^67^. The highest adsorption capacity observed at lowest temperature^59^. In this study optimum temperature selected was 27 °C.

Figure 11c displayed the interaction between temperature (C) and pressure (B). According to the experimental results, a rising pressure factor from 1 to 7.88 bar causes CO_2_ capacity to improves to 4.80 mmol/g. Khajeh and Ghaemi^68,69^ reported that molecular movement and rate of reaction increase in high pressure. They reported that the adsorbents with different pore sizes show various behavior. Consequently, the optimum pressure was chosen at 7.88 bar.

#### Model variation and condition optimization

The RSM-BBD as a numerical method was applied for optimization of operating conditions in the selected range factors by considering the standard error^28^. The precision between the purposed solutions and actual results was determined by the optimal solutions. The optimum conditions was obtained at adsorbent weight of 0.4 g, pressure of 7.88 bar, and temperature of 27 C. Under the optimum conditions, the highest CO_2_ capture was 4.80 mmol/g.

### Adsorbent regeneration

**Figure 12:**
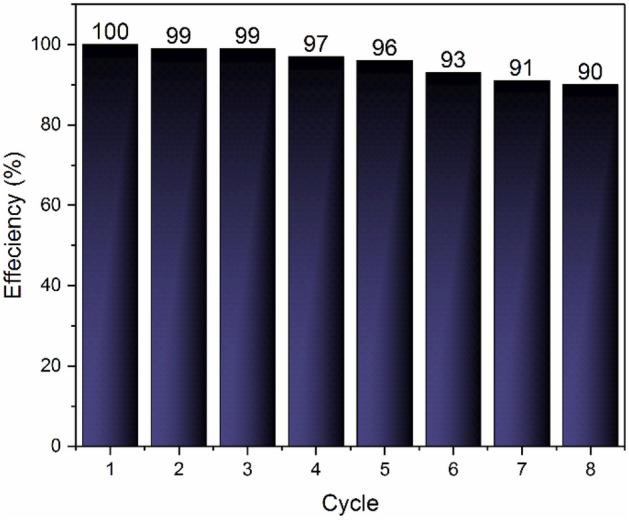
Recycling performance of Pg@GO for CO_2_ capture.

As an economical view, the regeneration of solid adsorbents is very important. The regeneration process is performed by three methods such as changing both of them resulting in a hybrid regeneration (VTSA/PTSA), changing the temperature (temperature swing; TSA), and changing the pressure (vacuum/pressure swing; VSA/PSA)^70,71^. In this study, the TSA method was used for regeneration of Pg@GO. At the end of each cycle, Pg@GO as a solid adsorbent was separated and regenerated in the oven at 40 °C for 10 h. As can be seen in Fig. 12, the adsorption capacity declined from 100 to 90% after 8 cycles. Because Pg@GO is economical and high-value adsorbent, it can be used in industrial gas adsorption applications.

### Comparison of the absorbent results with other absorbents

Table 7 compares the adsorption capacity of Pg@GO used in this research with other research that used GO as an adsorbent for CO_2_ capture. According to the results, it is clear that Pg@GO can adsorb CO_2_ at 7.88 bar and 300 C with the maximum capacity of 4.80 mmol/g. As Pg@GO has many active sites, it can adsorb CO_2_ molecules in the presence of low amount of solid adsorbent (0.4 g). At the end of the CO_2_ adsorption process, Pg@GO recycled eight times without decreasing the adsorption capacity.

**Table 7:** Comparison of operation condition and CO_2_ adsorption capacity.

Solid adsorbents	P (bar)	T (°C)	CO_2_ capacity (mmol/g)	References
KOH@GO–Fe_3_O_4_	9	298	6.812	^11^
GO–TiO_2_–Ag_2_O–Arg	10	273	1250	^72^
GO–TiO_2_–Ag_2_O	10	273	1185	^72^
polyethylenimine@GO	1	298	0.747	^73^
PEI-GO@ZIF-8	1	273	4.190	^74^
TiO2@GO	1	323	1.880	^75^
This study	7.88	300	4.800	–

## Conclusion

Carbon dioxide is one of the greenhouse gases that is produced via burning fossil fuels, and it has a negative impact on the environment. Pg@GO was synthesized as a solid adsorbent for CO_2_ capture. The RSM-BBD method was used to optimize operation conditions. The maximum CO_2_ adsorption capacity was 4.80 mmol/g at 0.40 g adsorbent weight, 300 K, and 7.88 bar. The Freundlich isotherm model has a good agreement with experimental data. Hence, the adsorption process is heterogeneous. According to kinetic model results, the Elovich model was able to describe CO_2_ adsorption data because of the highest R^2^ value and showed the interaction between CO_2_ molecules and the adsorbent’s surface is chemisorption. The negative value of ΔG° in the thermodynamic study proved that the process was exothermic and spontaneous. The regeneration of Pg@GO was tested in optimum conditions. The solid adsorbent was able to be reused eight times without a significant loss on the CO_2_ adsorption capacity.

## Data Availability

The data used and analyzed during the current study is available from the corresponding author on reasonable request.
